# Electrical semiconduction modulated by light in a cobalt and naphthalene diimide metal-organic framework

**DOI:** 10.1038/s41467-017-02215-7

**Published:** 2017-12-15

**Authors:** Evandro Castaldelli, K. D. G. Imalka Jayawardena, David C. Cox, Guy J. Clarkson, Richard I. Walton, Long Le-Quang, Jerôme Chauvin, S. Ravi P. Silva, Grégoire Jean-François Demets

**Affiliations:** 1 Departamento de Química, FFCLRP, Universidade de São Paulo, Av. Bandeirantes 3900, Ribeirão Preto, SP 14040-901 Brazil; 2 Advanced Technology Institute, University of Surrey, Guildford, Surrey GU2 7XH UK; 3 Department of Chemistry, University of Warwick, Coventry, CV4 7AL UK; 4 CIRE/DCM Université de Grenoble Alpes, CS 40700, Grenoble Cedex 9,, 38058 France

## Abstract

Metal–organic frameworks (MOFs) have emerged as an exciting class of porous materials that can be structurally designed by choosing particular components according to desired applications. Despite the wide interest in and many potential applications of MOFs, such as in gas storage, catalysis, sensing and drug delivery, electrical semiconductivity and its control is still rare. The use and fabrication of electronic devices with MOF-based components has not been widely explored, despite significant progress of these components made in recent years. Here we report the synthesis and properties of a new highly crystalline, electrochemically active, cobalt and naphthalene diimide-based MOF that is an efficient electrical semiconductor and has a broad absorption spectrum, from 300 to 2500 nm. Its semiconductivity was determined by direct voltage bias using a four-point device, and it features a wavelength dependant photoconductive–photoresistive dual behaviour, with a very high responsivity of 2.5 × 10^5^ A W^−1^.

## Abstract (web summary)

Photoactive and semiconducting metal-organic frameworks are desirable for electrical and photoelectrical devices, but remain rare. Here Demets and co-workers design a naphthalene diimide and cobalt based MOF with anisotropic electrical semiconductivity and a high responsivity of 2.5 × 10^5^ A W^−1^.

## Introduction

Metal–organic frameworks (MOFs) are a class of robust, microporous, crystalline organic–inorganic hybrid materials that have received much attention due to their myriad of properties and applications as well as owing to their inexpensive starting materials and simple synthetic routes^1–3^. MOFs may also feature large surface areas and inherit electronic and optical properties from their component metals and ligands. Despite the virtually infinite number of combinations between different ligands and metal centres, application of MOFs usually exploit their surface areas, mainly for gas adsorption^4^ and selective catalysis^5^. Their properties can also be tuned or altered depending on the characteristics of any adsorbed species and guest molecules^6, 7^. These coordination polymers are normally insulating materials with only a fraction being designed for electronic semiconductivity^8–10^, and almost none display any electrical semiconductivity for practical purposes^11, 12^. Examples of redox activity^13–16^, photoactivity^17, 18^ and electronic semiconductivity^19–21^ in MOFs can be found in the literature, but to a more limited extent than those focused on catalysis or gas adsorption. Most notably, Dincă et al. developed a series of MOFs with intrinsic semiconductivity^8–10^, while Nishihara also developed a series of electroactive, photoactive and semiconductive nanosheets of 2D coordination polymers with then record-high conductivities^22–26^. Another field where MOFs are currently being considered is solar cells, where they have been combined with well-known active semiconductors, such as TiO_2_ and carbon nanotubes^27, 28^. Despite their current limitation as intrinsic semiconductors, great progress has been made in recent years in the field of MOFs for electronics and photonics^29^.

1,4,5,8-naphthalene diimides (NDIs) are a class of organic compounds that form air-stable n-type organic semiconductors as thin films with high charge mobilities^30^. Their unique electrochemical and optical properties make them attractive materials for electronic applications, such as active layers in transistors^31^ and solar cells^32^, and as electron-transport layers in organic light-emitting devices^33^. Most of the electronic and optical properties of NDIs are governed by their frontier orbitals, mainly localised on the naphthalene core, and they usually experience small or minimal effects from their side chains^34^. In this sense, it is possible to choose suitable side chains to act as coordination points with no considerable influence on core properties. Redox-active MOFs with NDI ligands have been developed featuring the typical radical anion (NDI^•−^) and dianion (NDI^2−^) species with interesting optical characteristics^11, 13, 35^, and in-depth analysis has been carried out using computational methods^36^ and spectroelectrochemistry^37^. Their electron accepting nature may be exploited in extended networks to promote electron-hopping semiconduction between multivalent ionic metallic centres, such as (Ru, Fe, Os or Co)^II/III^ and others^19^. It is well known that NDIs may undergo light-induced reduction, forming radical anions and dianions, like negative polarons and bipolarons^38^.

Rationally combining NDI properties with a suitable metal may be key to yield semiconductive MOFs. The concept of efficient photoactive semiconducting MOFs by combining the stability, large surface areas and versatility of MOFs is crucial for the development of electrical and photoelectrical devices.

Here we report a unique, highly crystalline NDI-based MOF with semiconducting properties. We chose Co(II) as the metal centre to explore its suitable Pearson's hardness to *N*-coordinated ligands, and to exploit the electronic structure and spectroscopic features it delivers^39^, *N,N*′-bis(4-pyridyl)-1,4,5,8-naphthalene diimide (NDI-py) as the primary ligand and terephthalic acid (TpA) as a supporting ligand. This framework is labelled MOF-CoNDI-py-2, and its crystal structure and electronic properties are analysed in depth in order to understand its light sensitive electrical semiconductivity. Photocurrents and responsivities are assessed by direct measurements using a fabricated electronic device under a monochromatic light of different wavelengths. These results are evaluated in terms of anisotropy of semiconductivity and photoactivity, and are correlated with electronic absorption bands using a symmetry-adapted ligand field theory model.

## Results

### Synthesis of MOF-CoNDI-py-2

**Fig. 1:**
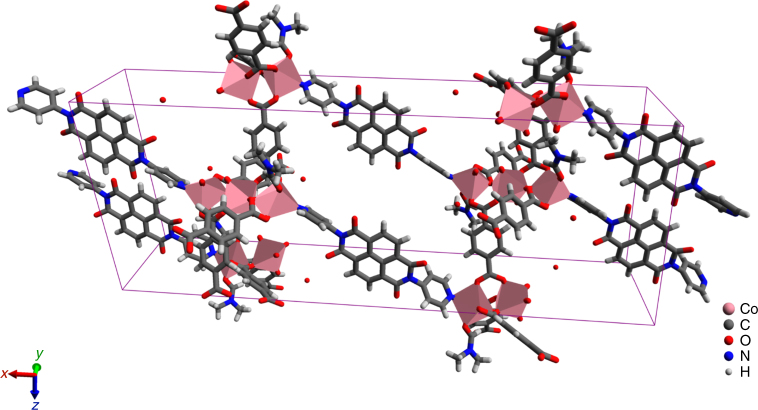
A representation of MOF-CoNDI-py-2 crystal structure including its unit cell obtained by single-crystal XRD. Pink = cobalt, grey = carbon, white = hydrogen, blue = nitrogen and red = oxygen. The unit cell belongs to a *C*2/*c* monoclinic space group, water was modelled at 1/4 occupancy and its hydrogens are not shown for clarity. The structure shows the NDI-py molecules oriented in a layered fashion. The distances between NDI-py entities are 9.43 Å along the *c*-axis, 9.57 Å along the *b*-axis and 11.10 Å through the metal cluster

**Fig. 2:**
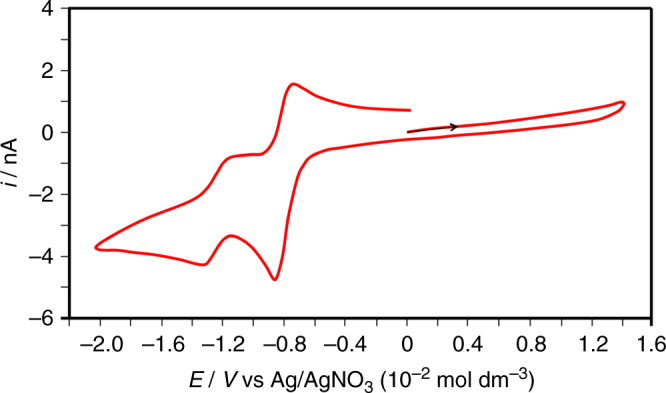
Solid-state cyclic voltammetry of MOF-CoNDI-py-2 in MeCN. A cavity microelectrode was used as working electrode, a platinum wire as auxiliary and Ag/AgNO_3_ (10 mmol dm^−3^, MeCN) as reference, in a 0.1 mol dm^−3^ [N(*n*-Bu)_4_](PF_6_) solution in acetonitrile at 50 mV s^−1^. The voltammogram shows characteristic naphthalene diimide reduction pairs at *E*_1/2_ = −0.79 and −1.25 V vs Ag/AgNO_3_, coupled with the Co(II)/Co(I) pair at −0.79 V

The compound was synthesised solvothermally in *N,N*-dimethylformamide (see 'Methods' section). MOF-CoNDI-py-2 forms purple leaf-like plates, which crystallised in the monoclinic space group *C*2/*c* and the contents of the unit cell are shown in Fig. 1. The asymmetric unit contains two crystallographically independent high spin Co(II) atoms, Co(1) and Co(2), whose valence were identified by EPR spectroscopy (Supplementary Fig. 1), electrochemically^40, 41^ (Fig. 2) and confirmed by bond–valence sums^42^ (Supplementary Table 1). There is one terephthalate strut on a general position and one that lies on an inversion centre. The NDI-py molecule sits on a twofold axis perpendicular to the plane of the molecule. An *N,N*-dimethylformamide is coordinated to Co(1), and a small amount of electron density was modelled as a solvent water at 1/4 occupancy. The diimide was modelled as disordered over two positions related by a small movement along the twofold axis enabled by a slight distortion in coordination of the pyridine to Co(1). The occupancy of the two components was fixed at 50:50, owing to the presence of the twofold axis. Planarity restraints were applied to some of the rings with additional restraints and constraints to ensure the expected geometry. The coordinated *N,N*-dimethylformamide molecule also showed severe thermal motion, and restraints were used to give it and the disordered components reasonable bond lengths, angles and thermal parameters. The large free volume in the unit cell is probably responsible for the uninhibited thermal motion of the struts of the framework, even at 100 K.

Further investigation into these solvent accessible voids gave comparable results of 2643.4 Å^3^ (33.0% of cell volume) using Olex2^43^ and 2557.6 Å^3^ (31.9% of cell volume) using Platon^44^, which is high, but on par with similar MOFs, and accounts for the extra solvent content seen by thermogravimetric analysis (Supplementary Fig. 2)^13, 45^.

Other Co(II) coordination polymers with carboxylate and *N*-donor ligands also contain the same trinuclear building unit of distorted octahedra, as in MOF-CoNDI-py-2^46–51^. Other MOFs with NDI-py as a ligand display rather different structures. For example, using Zn(II) as the metal centre, these frameworks usually contain a carboxylate-bridged paddle-wheel building unit, where the metal ions have a tetrahedral or a bidimensional square environment^45^. Previously reported MOFs using Co(II) with NDI-py ligands adopt linear coordinated polymeric structures significantly different from the structure reported here^52, 53^.

**Fig. 3:**
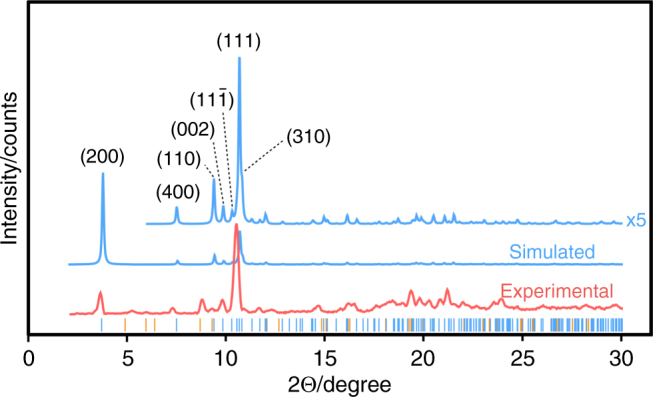
Experimental and simulated powder X-ray diffractograms of MOF-CoNDI-py-2 using a Cu source at *λ* = 1.54 Å. Below the curves are the calculated allowed peak positions as black vertical lines

The simulated powder X-ray diffraction pattern closely matches the experimental data set, as shown in Fig. 3, even with a significant temperature difference (simulated at 100 K and experimental at ca 300 K). The main difference between simulated and experimental data are the (200) and (111) peak intensities, assigned to unaccounted solvent molecules in the modelled crystal structure and to the flat morphology of the crystals, giving strong preferred orientation effects.

**Fig. 4:**
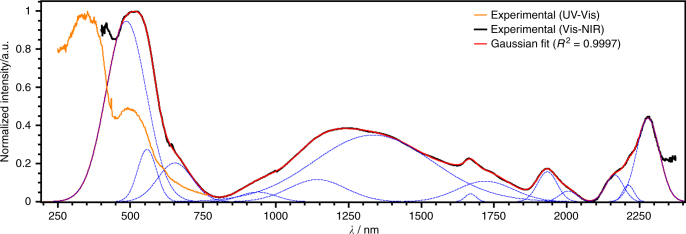
Comparison of electronic absorption spectra of MOF-CoNDI-py-2. The spectra were collected in the UV–Vis (orange) and Vis–NIR (black) range. Mathematically deconvoluted Gaussians for the Vis–NIR, only, are shown in blue dashed lines. The sum of all individual Gaussians is shown in red

**Fig. 5:**
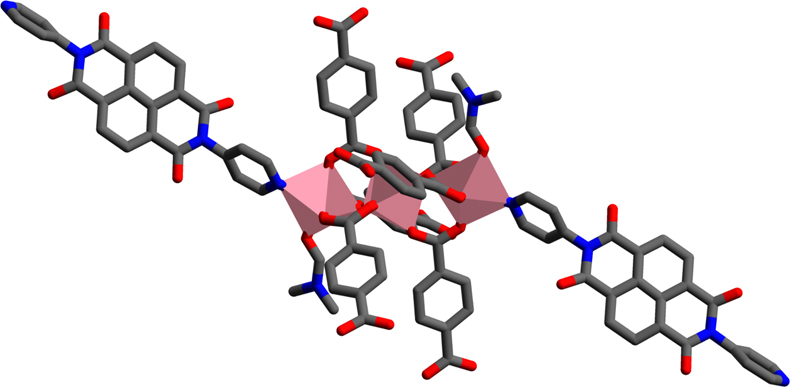
Spectroscopic unity of MOF-CoNDI-py-2. It is composed of three metal ions, six TpA anions, two NDI-py and two DMF molecules. Hydrogen atoms are not shown for clarity

MOF-CoNDI-py-2 has a broad and complicated absorption spectrum, as shown in Fig. 4. Its crystals absorb the whole radiation spectrum between 300 and 2350 nm except for two wavelengths at 800 and 2075 nm, and this fact is attributed to the low symmetry of the metallic centres. The trinuclear fragment depicted in Fig. 5 has a C_*i*_ symmetry, and there are two coordination environments for Co(II) ions. The first, in the middle, contains a metal ion in a quasi-octahedral site, while the others are equivalent and sit in a C_1_ symmetry. The resulting spectrum is composed of NDI-py’s S_0_–S_1_ transitions around 300–400 nm, a charge transfer band centred at 485 nm, and many weak *d–d* bands. Nine of these transitions are expected due to the previously mentioned low symmetry of Co(II) atoms, leading to completely non-degenerate Russell–Saunders terms. In comparison with simple O_*h*_ ligand fields, the triply degenerate states—T_1*g*_ and T_2*g*_—will split in 3 A_*g*_ terms in a C_*i*_ coordination symmetry. The observed bands have estimated absorptivities of 10^3^ L mol^−1^ cm^−1^, one order of magnitude higher than expected. These observations are probably due to the lower metal symmetry and to metal–ligand *π*-bonding^54^, as all coordinated atoms from ligands are *sp* ^2^ in nature. The most intense band in the Vis–NIR spectrum is assigned to a metal-to-ligand charge transfer (MLCT) as the NDI-py ligand has a strong *π*-acceptor character, demonstrated by its known reduced species in low electrochemical potentials^34, 55^, at −0.83 V vs Fc/Fc^+^ (ferrocene/ferrocinium). Solid-state cyclic voltammetry, shown in Fig. 2, revealed two redox pairs at −0.79 and −1.25 V vs Ag/AgNO_3_, a characteristic of naphthalene diimides^33, 55^, and this allowed us to estimate the LUMO energy of −3.9 eV, suggesting it is mainly NDI-py in character (see Supplementary Fig. 3).

### Photoelectrical characterisation of MOF-CoNDI-py-2

**Fig. 6:**
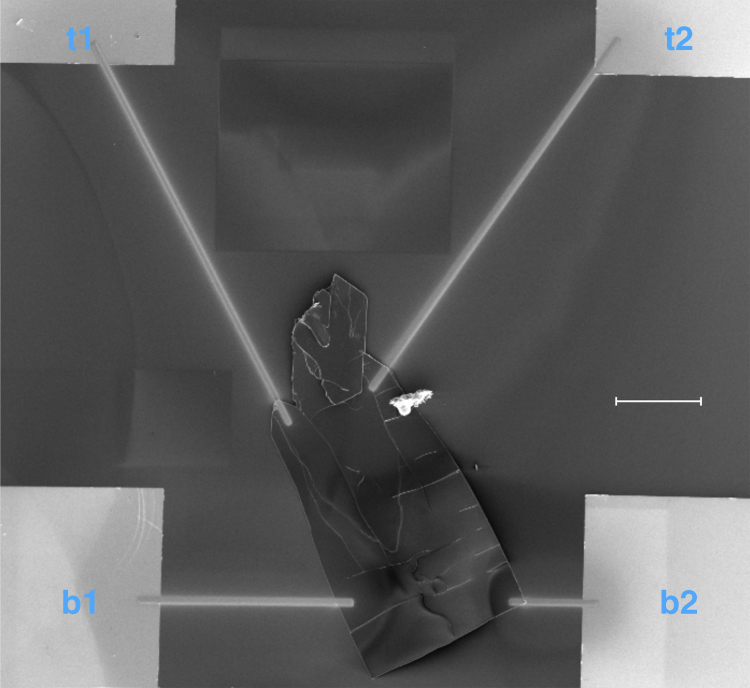
SEM image of the device used in all electrical and photoelectrical characterisations. It consists of a *p*-Si/SiO_2_ wafer onto which a MOF-CoNDI-py-2 single crystal is connected to Au pads, labelled **t1**, **b1**, **t2** and **b2**, by deposited Pt electrodes. Scale bar = 100 μm

**Fig. 7:**
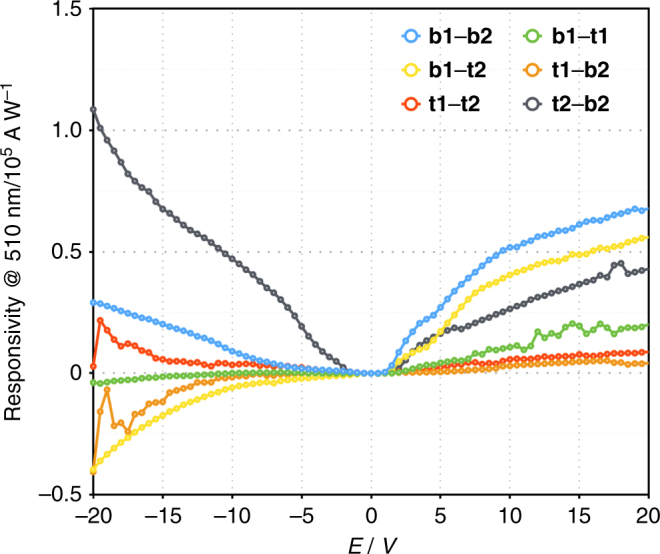
MOF responsivity under monochromatic light illumination. Responsivity measurements of the MOF device probing all directions under a 510 nm monochromatic light

We fabricated the device shown in Fig. 6 for the photoelectrical characterisation of MOF-CoNDI-py-2. The material was analysed by probing every pair of pads, identified as **t1**, **t2**, **b1** and **b2**, by applying a sweeping voltage bias under monochromatic light. We studied the material under strong absorption bands (350, 380, 460, 510, 550 and 630 nm) and weaker bands (700 and 800 nm) in the reflectance spectrum of the framework, as noted in Fig. 4. The crystal face parallel to the substrate, where all four electrodes are connected, is the (100) plane, which is orthogonal to the Co⋯NDI-py bond and parallel to the Co⋯TpA bonding plane (see Supplementary Fig. 4). The resulting current vs voltage curves are highly anisotropic and wavelength-dependent. The whole set can be found in the Supplementary Figs. 5–8. Here we show in Fig. 7 the responsivity measurements of all probed directions when excited with a 510 nm wavelength. In this case, this wavelength is mainly charge-transfer in character, with a low contribution of *d–d* transition. The highly asymmetric nature is indicative of a metal–semiconductor contact, or Schottky, effect. The metal–ligand interactions forms nanoscale Schottky diodes throughout the crystal, providing a high built-in field that efficiently dissociates the excitons generated under illumination, and this is further supported by the very high photocurrents observed discussed below.

## Discussion

**Fig. 8:**
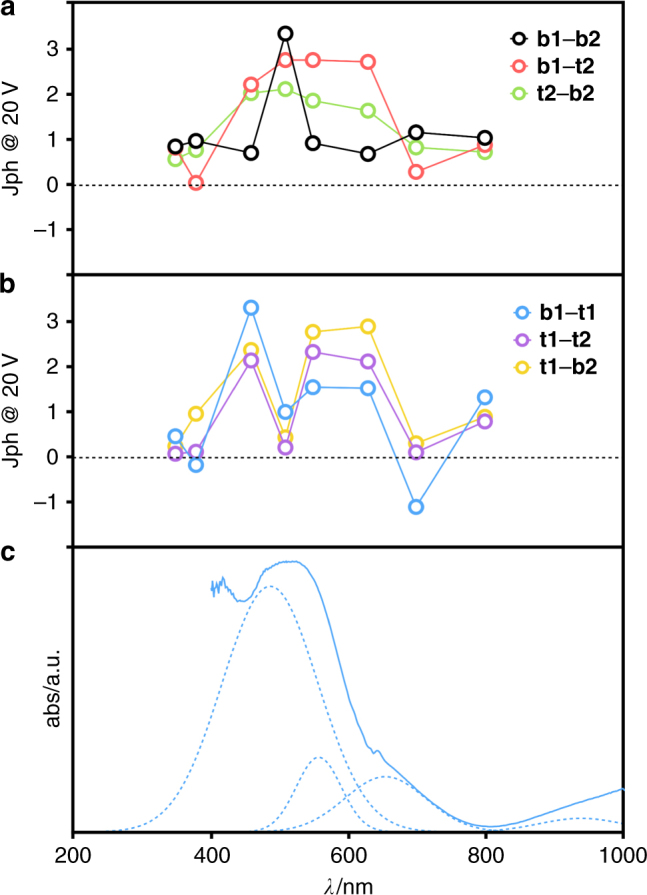
Comparison of photoresponse intensities at 20 V with the deconvoluted absorption spectrum of MOF-CoNDI-py-2. **a**, **b** Photoresponse intensities at 20 V. **c** Electronic absorpotion spectrum of MOF-CoNDI-py-2

The crystal displays photoresponse intensities (Jph) proportional to band intensities at that wavelength, as shown in Fig. 8, at which the maximum outputs coincide with the MLCT band. We believe that the conduction mechanism involves a charge transfer from the metal centre to the strong *π*-acceptor NDI-py, promoting hole transport through the Co⋯TpA direction while electrons are transported by the NDI-py direction. While the distance between NDI-py unities are unusually high for charge transport, these are not far off from similar structures used as high-mobility active materials in transistors (up to 8.3 Å)^30^. Excitation at the MLCT band further improves this mechanism by promoting a charge injection from metal to diimide. All orientations follow the same trend however directions involving **t1** display a sharp Jph decrease at 510 nm, and this is attributed to the fact that **t1** sits on a different crystal layer than all the other pads. This introduces a significant *z*-axis component, orthogonal to the (100) crystal face and through the Co⋯NDI-py bond, to the electron path not experienced in this degree by any other pad, and this additional crystal direction in all combinations involving **t1** leads to a slightly distorted behaviour. Responsivities at 20 V were at very high values of 2.5 × 10^5^ A W^−1^ (**t2**–**b2** at 800 nm), and followed the same trend as Jph although they were unexpectedly higher at 800 nm, which indicates the crystal’s capacity to harvest photons efficiently even at wavelengths with low absorptivity. In Supplementary Table 2, we compare the observed responsivity of MOF-CoNDI-py-2 to other materials. Measurements of the photocurrent under different illumination intensities (20–100 mW cm^−2^, see Supplementary Figs. 9 and 10) indicate a linear response under an applied bias of ±20 V, which equates to a bulk electric field of less than 0.1 V µm^−1^. The observation of a linear behaviour over such a wide illumination intensity indicates no variation in both the external as well as internal quantum efficiencies for the range of illumination intensities studied. Furthermore, the linear photoresponse under such high illumination intensities also eliminates thermal effects as the origin for the high photoresponses observed^56^. We believe part of the reason for these very high responsivities are the high surface areas, each with an electric field across the device. As a result the depleted active region can act upon the total optical flux, giving high responsivity with large sensory volumes. The high responsivities observed here are indicative of an efficient exciton dissociation mechanism. Efficient exciton dissociation requires the presence of strong built-in fields, which far exceed the bulk fields applied to the device here. Such strong nanoscale fields are expected due to the interaction between the metal ion and the organic ligand. We note that there is evidence of high photoactivity in highly crystalline MOFs^57^. While exciton dissociation as a result of a Schottky contact at the metal/MOF interface is also possible, this is considered to be highly unlikely as the shading effect by the metal reduces the photocurrent generation. Except for the **t1**–**b1** direction, the device displayed positive Jph and responsivities at positive bias while at negative bias, with the exception of the **t2**–**b2** direction, the device had a dual photoconductive–photoresistive behaviour depending on the incident wavelength.

Current vs voltage measurements carried out in darkness indicate asymmetric characteristics for the MOF crystal with Pt contacts. This is ascribed to the anisotropic charge semiconduction and different charge transport properties along the Co⋯NDI-py direction as opposed to the direction perpendicular to aromatic rings. The electrical characteristics are expected to be further influenced by the different electronic properties at the crystal faces at which the Pt metal comes into contact with the MOF crystal^58–60^.

Observation of the photocurrent characteristics under negative bias for monochromatic illumination reveals a mixture of positive and negative photocurrents, as opposed to the positive only photocurrent values expected. This is in contrast to the photoresponse under white light bias measurements where there does not appear to be a mix of positive and negative responses. It is noted here that there is a significant difference in the illumination intensities used under monochromatic response (nW cm^−2^) measurements as opposed to white light measurements (mW cm^−2^). As a result, the dark current density of the material under different configurations plays a significant role in the sign of the photoresponse observed, especially at lower intensities as used in observing the monochromatic response. Light intensities at which the photocurrent generated is lower than the dark current density results in a negative photocurrent response. This effect is further amplified by the anisotropic semiconducting properties of the crystal, the optical absorption coefficients under different wavelengths, as well the properties of the interfacial contacts between the metal and the crystal faces. However, these factors are less likely to affect the photocurrent response under higher light intensities as those used for the measurements carried out under white light due to the significantly higher photocurrents generated.

An interesting feature of the MOF crystals synthesised in this work is the observation of both photo-conductance (increase in the current under illumination) and inverse photo-conductance (or photo resistance, reduction of current under illumination). We note that previously, Nakanishi et al. have reported both photo and inverse photo-conductance characteristics which was ascribed to the combination of a self-assembled monolayer and the metal nanoparticle used^61^. One possible reason for the observed inverse photo-semiconduction in this work is the ability of the metal centres to act as charge trap sites due to their oxidation state. This follows from the expected energy diagram formed upon complexation, as the HOMO level will have a major contribution from the ligands, thus being more localised in the organic components while the SOMO will have a large Co character^39^. This energy alignment favours hole trapping in Co(II) ions. However interestingly, the crystal does not appear to indicate inverse photo-semiconductivity for the same group of wavelengths probed for the different combinations of electrical contacts, indicating that both the bulk and the contact properties are likely to play an important role in the observed photo and inverse photo-semiconduction characteristics.

Despite its anisotropy that lead to different conductivity values, the crystal conducts electricity in all the directions that could be measured by our four-point probe apparatus, showing the whole structure is conductive, and that electrons would not be transferred between the metallic centres exclusively by NDI moieties as one could expect, due to its semiconductive properties and conjugation that favours charge delocalisation. The results suggest that long-distance electron transfer and terephthalate-mediated electron transfer may happen as well.

In summary, we have synthesised and fully characterised MOF-CoNDI-py-2, a naphthalene diimide-based metal–organic framework featuring high crystallinity, electrochemical activity, electrical semiconductivity and photoactivity. The observed anisotropic electrical semiconduction also features a photoresistive–photoresponsive dual behaviour and one of the highest responsivities ever reported, which opens a new horizon in the development of MOF devices.

## Methods

### Synthesis of NDI-py

The synthesis of *N,N*-bis(4-pyridyl)-1,4,5,8-naphthalene diimide is described in the literature^62^, where we followed an adapted route^63^, using 503.7 mg (1.88 mmol) of 1,4,5,8-naphthalic dianhydride (Aldrich), 543.4 mg (5.77 mmol) of 4-aminopyridine (Acros) in ca 2 g of molten imidazole (Vetec), heated in an oil bath for 30 min and allowed to cool naturally to room temperature. The resulting solid was treated with a mixture of H_2_O:EtOH:HNO_3_ 10:10:1 (v/v, Synth) and filtered, washed again with the same solution and then sequentially with H_2_O, EtOH (Synth) and Et_2_O (Synth). The resulting product was a beige solid with 94% yield, 742.1 mg. This solid was then recrystallised in DMF with 47% yield. ^1^H-NMR (CF_3_COOD, ppm): 9.11 (4H, d, *J* = 6.5 Hz); 9.03 (4H, s); 8.37 (4H, d, *J* = 6.5 Hz). ^13^C-NMR (CF_3_COOD, ppm): 164.8; 155.0; 145.1; 135.0; 131.4; 129.7; 128.8. FTIR (KBr, cm^−1^): 3065, 3035, 1716, 1674, 1590, 1580, 1501, 1491, 1448, 1414, 1350, 1251, 1214, 1198, 1148, 1123, 985, 863, 827, 767, 751, 718, 623, 529. MS (*m/z*): 421 [M + H]^+^, 211 [M + 2 H]^2+^. See Supplementary Figs. 3 and 11–16 and Supplementary Note 1 for reaction scheme, NMR, UV–Vis, FTIR and electrochemical data.

### Synthesis of MOF-CoNDI-py-2

The metal–organic framework was synthesised by a solvothermal route, dissolving 0.24 mmol of NDI-py, 0.48 mmol of TpA and 0.48 mmol of Co(NO_3_)_2_⋅6H_2_O in 50 mL of *N,N*-dimethylformamide. This solution was then transferred to a polytetrafluoroethylene vial within a stainless steel casing. The vessel was heated at 80 °C for 48 h and allowed to cool to room temperature. Crystals were collected by filtration and washed thoroughly with *N,N*-dimethylformamide. Yield, based on NDI-py ligand, was 50%. See Supplementary Tables 2 and 3 for bond–valence sums and crystal data, and Supplementary Figs. 1, 2 and 16 for EPR, TGA and FTIR data.

### X-ray diffraction

Powder samples were dried in an oven at 300 °C for 3 h and analysed in a Siemens D5005 diffractometer with a Cu wavelength, using K*α*, at 0.02 °C min^−1^. For the single crystal analysis, a suitable crystal of MOF-CoNDI-py-2 was selected and mounted on a Mitegen loop with Fromblin oil and placed on a Bruker-Nonius APEX II CCD diffractometer equipped with a Bruker-Nonius FR591 rotating anode at the UK's National Crystallography Service, University of Southampton^64^. The crystal was kept at 100(2) K during data collection. Using Olex2^43^, the structure was solved with the ShelXS structure solution programme using Direct Methods and refined with the ShelXL refinement package using Least Squares minimisation^65^. CCDC 1517923 contains the supplementary crystallographic data for this paper. These data can be obtained free of charge from the Cambridge Crystallographic Data Centre.

### Crystal face indexing

A suitable crystal was selected and mounted on a glass fibre with Fromblin oil, as shown in Supplementary Fig. 17, and placed on a Rigaku Oxford Diffraction SuperNova diffractometer with a dual source (Cu at zero) equipped with an AtlasS2 CCD area detector. The crystal was kept at 100(2) K during data collection. Data were recorded with CuK_α_ radiation and frames, data processing and indexing was performed with the CrysAlisPro 1.171.38.43f software (Rigaku Oxford Diffraction, 2015).

### Simulated powder X-ray diffraction

The X-ray powder pattern was calculated using CCDC's (Cambridge Crystallographic Data Centre, University of Cambridge, UK) Mercury software, using a Cu source (*λ* = 1.54056 Å), 0.02 degree step and 0.1 units of 2Θ of full-width at half-maximum.

### EPR spectroscopy

X-band EPR spectra were recorded at 7 and at 25 K with a EMX Bruker spectrometer equipped with a ER4116DM Bruker cavity, an Oxford Instrument Cryostat (ESR900) and a Bruker temperature controller (ER4131VT).

### Electronic absorption spectra

Electronic absorption spectra of MOF-CoNDI-py-2 crystals were collected by diffusive reflectance spectroscopy using an Analytical Spectral Devices FieldSpec 3 spectrophotometer for the Vis–NIR region (350–2500 nm) and an Ocean Optics DH-2000-Ball for the UV–Vis region (250–800 nm), both using MgO (Synth) as reference.

### Fourier transform infrared spectra

FTIR spectra of NDI-py, TpA and MOF-CoNDI-py-2 were acquired in a Shimadzu IRPrestige-21 Fourier transform infrared spectrophotometer using KBr pellets.

### Polarised Raman spectra

Data were acquired using a Witec Alpha 300 R with a 532 nm 7.5 mW cm^−2^ Nd:YAG laser. Polarisers at the source and detector were positioned and rotated relative to one another at the indicated angles. Crystals of MOF-CoNDI-py-2 were deposited on a glass slide from a DMF suspension and dried on a hot plate. See Supplementary Figs. 18 and 19, and Supplementary Note 2 for spectra and discussion.

### Electrochemistry

Cyclic voltammetry of NDI-py was recorded with an Autolab PGSTAT30 Potentiostat/Galvanostat at 25 mV s^−1^ using a 1 mmol dm^−3^ solution in DMF (Aldrich) containing 10 mmol dm^−3^ of [N(*n*-Bu)_4_](PF_6_) (Aldrich) as supporting electrolyte, a glassy carbon electrode (*ϕ* = 1 mm) as working electrode, a 1 cm^2^ Pt counter electrode, a silver wire as pseudo-reference electrode and ferrocene (Aldrich) as internal reference.

### Solid-state electrochemistry

Cyclic voltammetry of MOF-CoNDI-py-2 was recorded at 50 mV s^−1^ in a EG&G model 173 Potentiostat/Galvanostat equipped with a PAR model universal programmer and a PAR model 179 digital coulometer using a cavity microelectrode (*ϕ* = 50 μm, *d* = 25 μm and *V* = 1 × 10^−8^ cm^3^) in a three-electrode arrangement, with an Ag wire as counter electrode, Ag/AgNO_3_ (Aldrich, 1 × 10^−2^ mol dm^−3^ in MeCN) as reference electrode and 0.1 mol dm^−3^ [N(*n*-Bu)_4_](PF_6_) (Aldrich) in MeCN (Aldrich) as supporting electrolyte.

### Electron microscopy

Scanning electron microscopy images of the fabricated devices were taken using a FEI Nova Nanolab 600.

### Electronic device

A 200 nm SiO_2_ layer was deposited onto a *p*-Si substrate via plasma-enhanced chemical vapour deposition (PECVD). Gold electrodes were deposited onto this Si/SiO_2_ substrate by lithography, with a pre-layer of titanium for adherence. These were cleaned with ultrasound bath in acetone for 5 min and dried over nitrogen flow. MOF-CoNDI-py-2 crystals were then deposited from acetone suspensions and connected to the Au electrodes by Pt electrodes, via vapour deposition using the sublimating compound trimethyl(methylcyclopentadienyl)platinum(IV) (Aldrich, 98%) as source. A crucible containing this compound, within the Focused Ion Beam (FIB) system in a FEI Nova Nanolab 600 dual beam system, is slowly heated to 50 °C and the vapour is directed to the desired location through a needle.

### Electrical characterisation

Current vs voltage curves of the fabricated devices were determined in air using two spring loaded probes using a Keithley 2400 in a two-point probe arrangement. Photoresponses were measured using a Bentham PVE300 Quantum Efficiency with the same devices.

### Photoconductivity measurements

The photoconductivity measurements were carried out using a Bentham PVE300 system consisting of xenon quartz halogen lamps. The appropriate wavelength was selected using the built-in monochromator in the system. The device was placed inside a dark box, which consists of ports connected to the lamps. The devices were connected to a Keithely 2400 operated in a four-wire configuration and two spring loaded probes were used to contact the lithographically patterned contact pads. The temperature of the device was maintained at 25 ± 0.5 °C using a built-in temperature controlling stage in the setup. The output intensities of the two lamps are given in Supplementary Table 4. The responsivity was calculated by using Eq. (1).

1 $${\mathrm{Responsivity}} = \frac{{I_{it} - I_d}}{{W \times \left( {\frac{{A_c}}{{A_s}}} \right)}}$$

In the equation above, *I*_it_ is the total current under illumination, *I*_d_ is the dark current, *W* is the illumination intensity, *A*_c_ is the crystal area and *A*_s_ is the area of the spot size where the crystal is placed. In order to minimise errors in estimating the crystal area, the SEM micrograph was used to calculate the crystal area.

### Thermogravimetric analysis

TGA and DTG analysis were carried out in a TA Instruments SDT 2960 Simultaneous DTA-TGA Thermal Analyst 2100 in oxidising atmosphere from room temperature to 1000 °C at 10 °C min^−1^

### Data availability

CCDC 1517923 contains the crystallographic data for MOF-CoNDI-py-2. The data can be obtained free of charge from the Cambridge Crystallographic Data Centre via www.ccdc.cam.ac.uk/getstructures. All the other data are available from the authors on reasonable request.

## Electronic supplementary material

Supplementary Information
