# Supplementary material for: Electrical semiconduction modulated by light in a cobalt and naphthalene diimide metal-organic framework
Source: Nat Commun. 2017 Dec 15;8:2139. doi: 10.1038/s41467-017-02215-7 (PMC5732256; doi:10.1038/s41467-017-02215-7)
Supplement: Supplementary file 1 — Supplementary Information [file 41467_2017_2215_MOESM1_ESM.pdf]

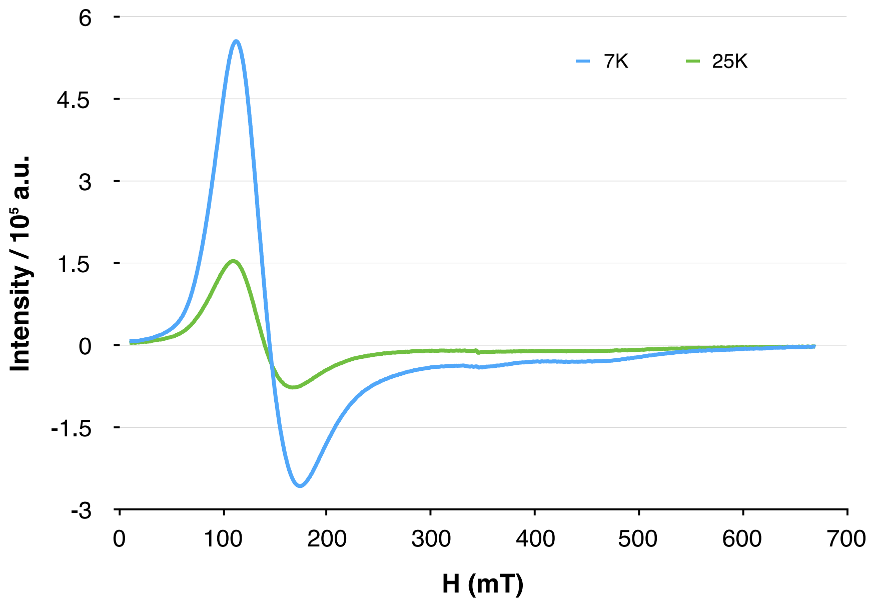

Supplementary Figure 1 | MOF EPR spectra. X-band electron paramagnetic resonance spectra of a powder sample of MOF-CoNDI-py-2 recorded at 7 K and 25 K (P = 6.533 mW), recorded with a EMX Bruker spectrometer equipped with a ER4116DM Bruker cavity, an Oxford Instrument Cryostat (ESR900) and a Bruker temperature controller (ER4131VT). The system shows a broad EPR signal without hyperfine pattern characteristic of a Co(II) (d^7) ion in the high spin state ($S = {}^3/_2$). The effective g values (≈ 4) and the Zero Field Splitting D > 1.5 cm^{-1} are in lines with those of other complexes of cobalt(II) with oxygen and nitrogen-donor ligands.[1,2]

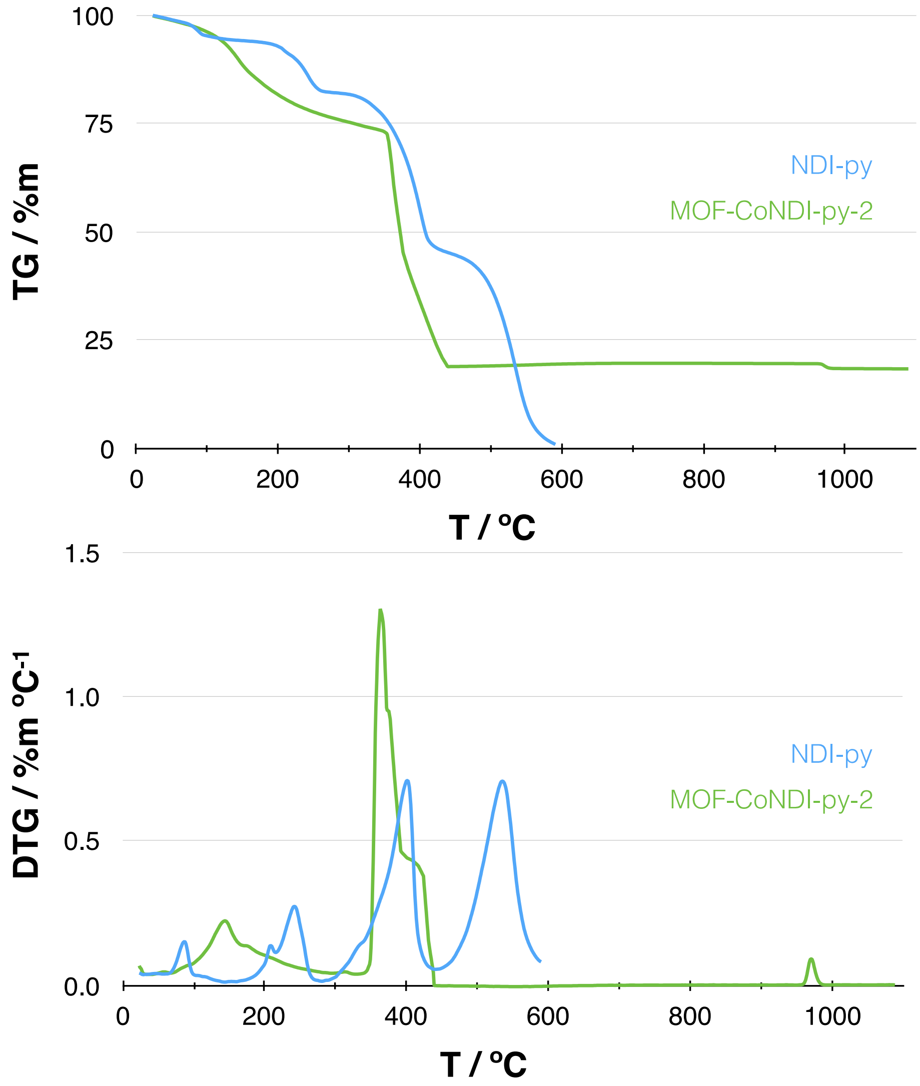

Supplementary Figure 2 | TGA and DTG curves of components in O_2 atmosphere. TGA and DTG analysis of NDI-py, TpA and MOF-CoNDI-py-2 in O_2 atmosphere. Terephthalic acid decomposes at temperature ranging from 250 ºC to 360 ºC. NDI-py loses solvent molecules from 60 ºC up to 100 ºC and undergoes a mutli-step decomposition process beginning at 180 ºC until its total decomposition at 600 ºC. MOF-CoNDI-py-2 loses adsorbed DMF around 110 ºC and coordinated/included DMF from 150 ºC to 210 ºC. Consecutive important mass losses occur between 340 ºC and 440 ºC that were assigned to the decomposition of TpA and NDI-py organic ligands, and of nitrate counter ion as well. Calculated NDI-py:TpA:DMF:Co:$NO3^-$ stoichiometry:4:9: 4:14:5; found: 4:9:5.3:14.1:4. The DMF excess is due to adsorbed solvent molecules.

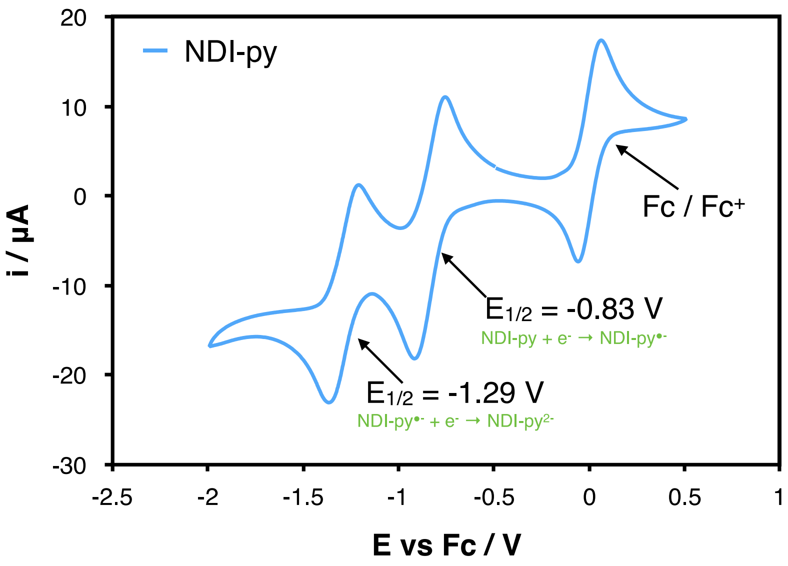

Supplementary Figure 3 | Cyclic voltammetry of NDI-py. Cyclic voltammogram of NDI-py (0.1 mmol dm^{-3}) in 10 mmol dm^{-3} $[N(n\text{-}Bu)_4](PF_6)$ (DMF). The two reduction process corresponding to the formation of NDI-py$^{\bullet-}$ and NDI-py^{2-}. The LUMO energy level was estimated by $E_{LUMO} = E_{Fc} + E_{1/2}(NDI\text{-}py/NDI\text{-}py^{\bullet-})$, with E_{Fc} being the LUMO level of ferrocene in the vacuum, determined as 4.8 eV[3]. Using the formula $E(Fc/Fc^{+}) = E(Ag/AgNO_3) - 87\ mV$, the LUMO level of NDI-py is estimated at -3.97 eV. Using the same procedure for MOF-CoNDI-py-2, and considering the estimated LUMO level of MOF-CoNDI-py-2 is -3.92 eV.

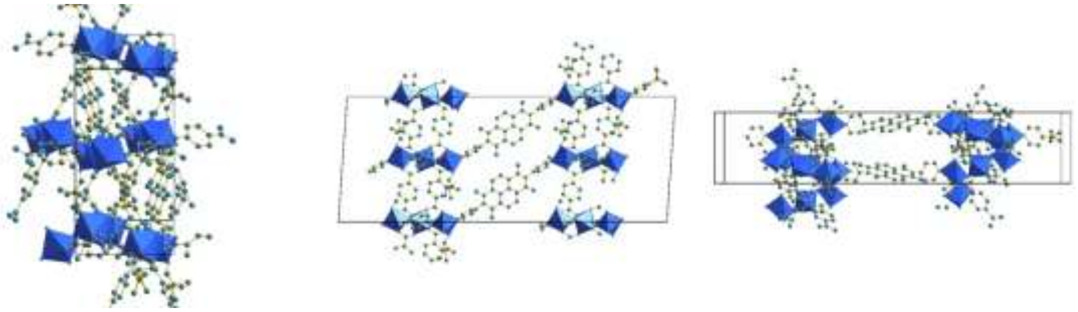

a) a-axis view. **b)** b-axis view. **c)** c-axis view.

Supplementary Figure 4 | Crystal profiles. Different points of view perpendicular to the (100) crystal plane of MOF-CoNDI-py-2.

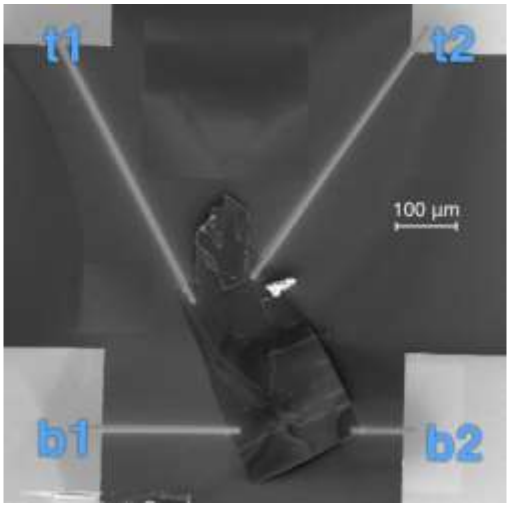

Supplementary Figure 5 | Electrical characterization device. Device used in all electrical measurements in air. Gold pads are labelled **t1**, **b1**, **t2** and **b2**, connected to the MOF-CoNDI-py-2 crystal through Pt electrodes.

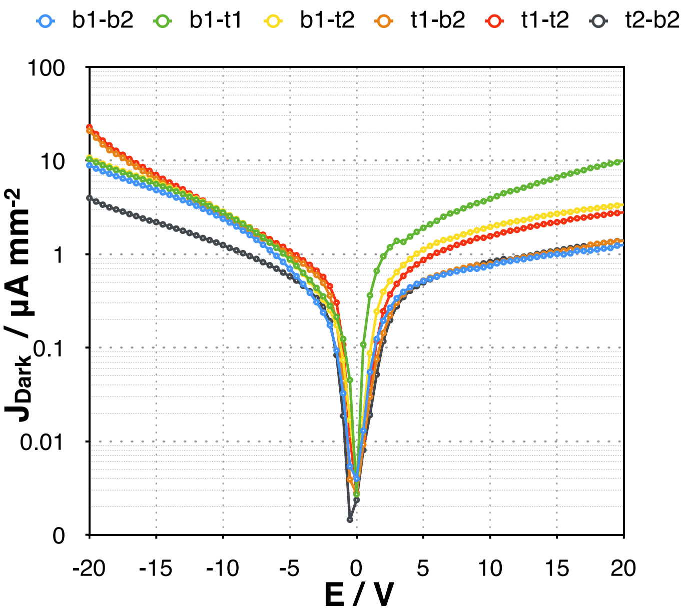

Supplementary Figure 6 | Dark current curves. Dark currents of MOF-CoNDI-py-2 for every pair of pads using the labels indicated in Supplementary Figure 5.

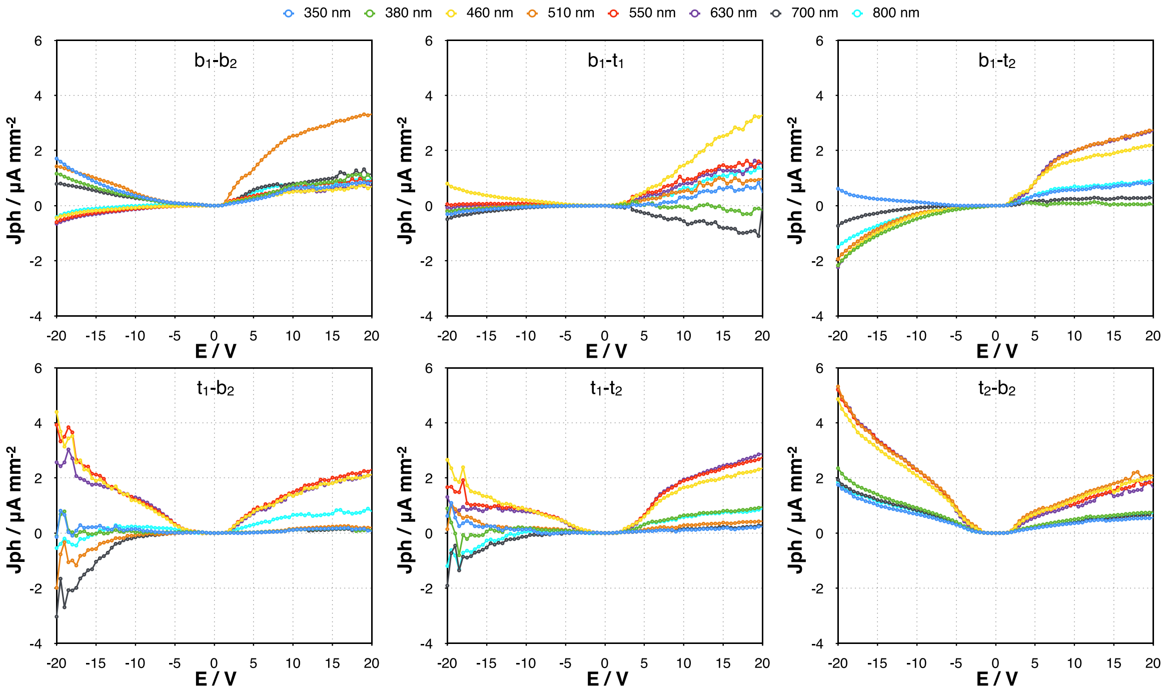

Supplementary Figure 7 | Photoconductivity measurements. Photoconductivity (Jph) measurements of MOF-CoNDI-py-2 for every pair of pads using the labels indicated in Supplementary Figure 5.

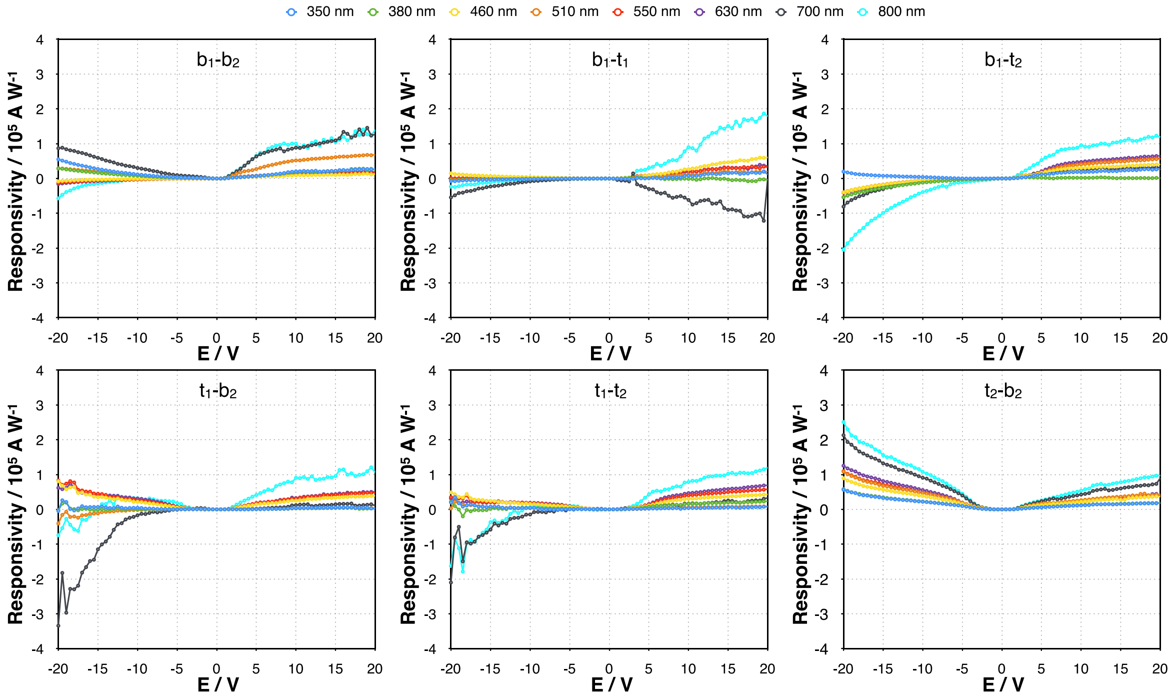

Supplementary Figure 8 | Responsivity measurements. Responsivity measurements of MOF-CoNDI-py-2 for every pair of pads using the labels indicated in Supplementary Figure 5.

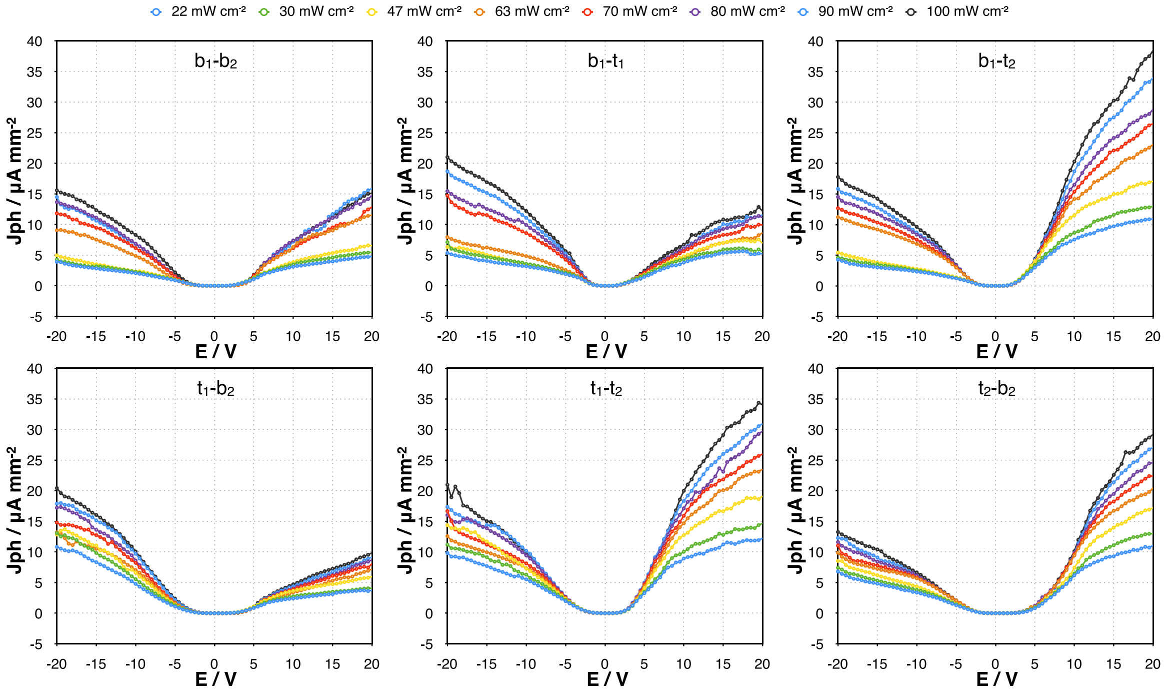

Supplementary Figure 9 | Influence of illumination intensity on photocurrent. Photoconductivity (Jph) measurements of MOF-CoNDI-py-2 for every pair of pads using a variable illumination intensities, studied for every pair of pads (Supplementary Fig. 5) using a modulated white light.

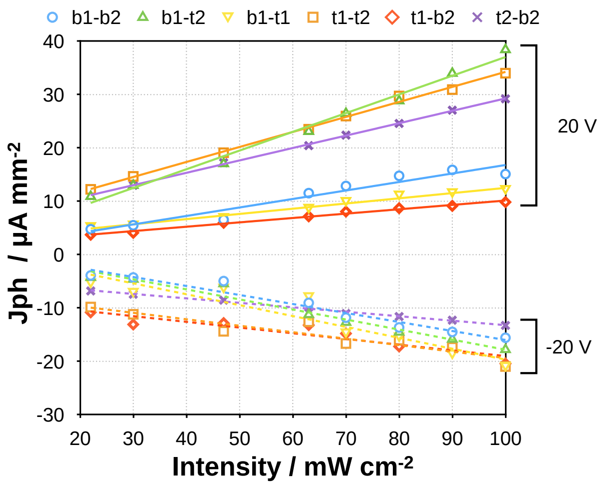

Supplementary Figure 10 | Photocurrent measurements. Trend lines of photoconductivities of MOF-CoNDI-py-2 at +20 V and -20 V using variable illumination intensities, for every pair of pads. The output intensity of the two lamps used in the photoconductivity measurements were measured at several wavelengths **(nm; pW mm^{-2})**: (350;31); (380;40); (460;54); (510;49); (550;47); (630;41); (700;9); (800;7).

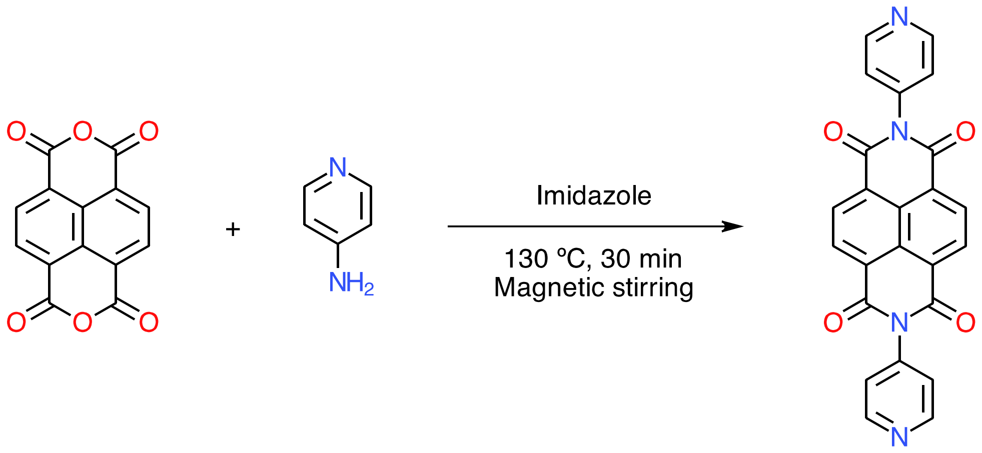

Supplementary Figure 11 | Naphthalene diimide synthesis. Scheme for the synthesis of *N,N*-bis(4-pyridyl)-1,4,5,8-naphthalene diimide (NDI-py).

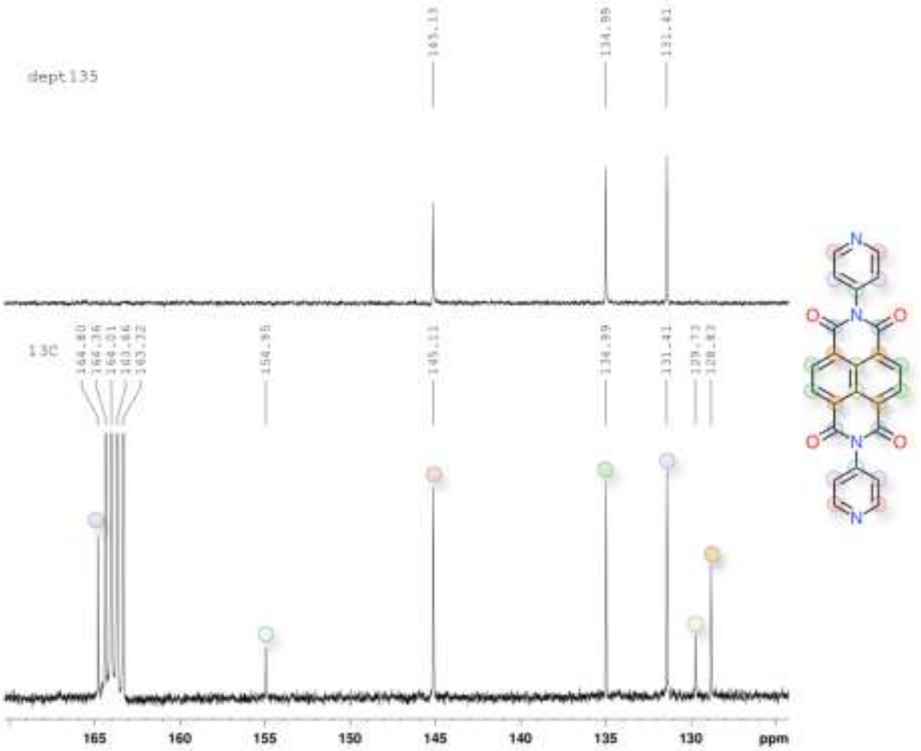

Supplementary Figure 12 | DEPT 135 and carbon NMR for NDI-py. Dept135 (top) and ^{13}C-NMR (bottom) spectra of NDI-py in CF_3COOD (500 MHz). ^{13}C-NMR (CF_3COOD, ppm): 164.8; 155.0; 145.1;135.0;131.4; 129.7; 128.8.

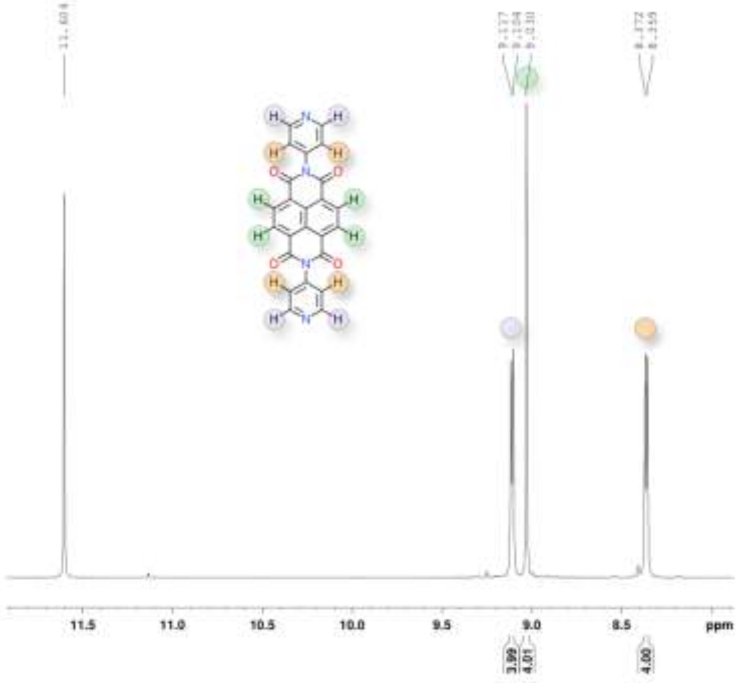

Supplementary Figure 13 | NDI proton NMR. ^{1}H-NMR spectrum of NDI-py in CF_3COOD (500 MHz). ^{1}H-NMR (CF_3COOD, ppm): 9.11 (4H, d, J = 6.5 Hz); 9.03 (4H, s); 8.37 (4H, d, J = 6.5 Hz).

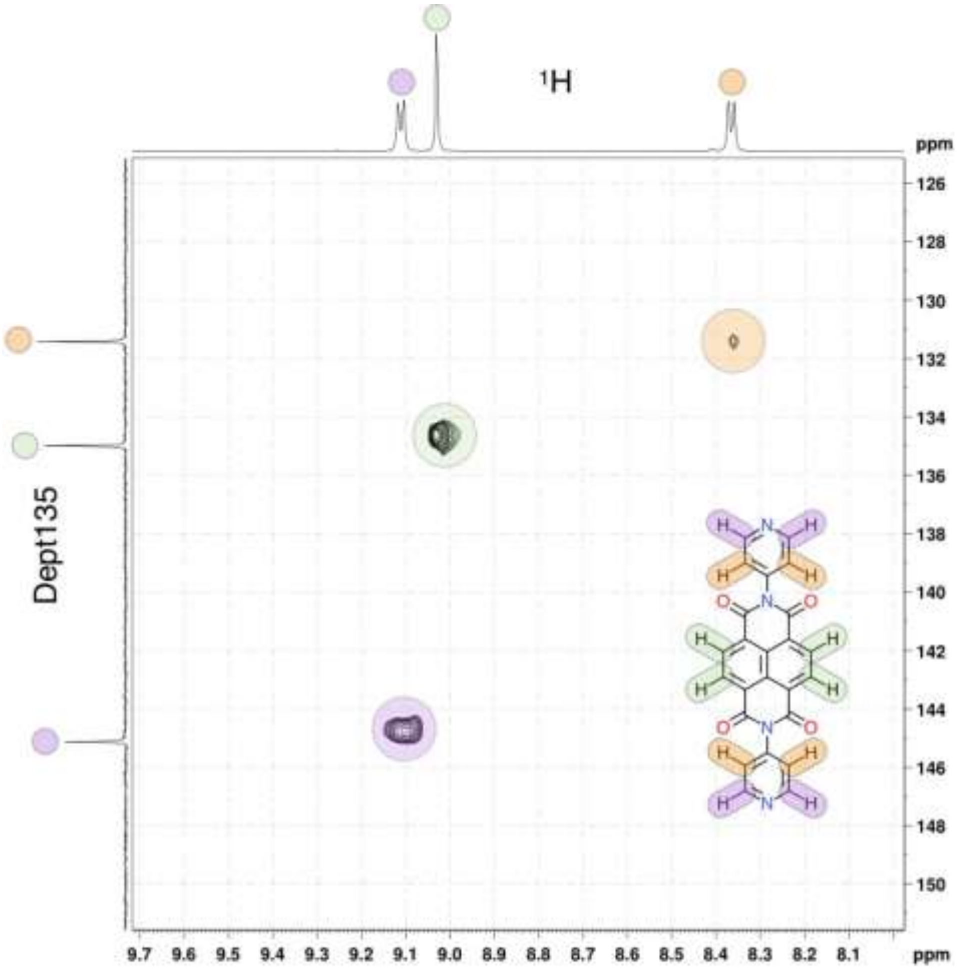

Supplementary Figure 14 | NDI-py HMBC NMR spectrum. Heteronuclear Multiple Bond Correlation NMR spectrum of NDI-py in CF_3COOD (500 MHz). It correlates Dept135 (featuring only C-atoms with C–H bonds, in this case) with ^{1}H-NMR, indicates that the hydrogens at δ = 9.11 ppm are coupled with the carbons at δ = 145.1 ppm, while the hydrogens at δ = 9.03 ppm are coupled with the carbons at δ = 135.0 ppm and the hydrogens at δ = 8.37 ppm are coupled with the carbons at δ = 131.4 ppm.

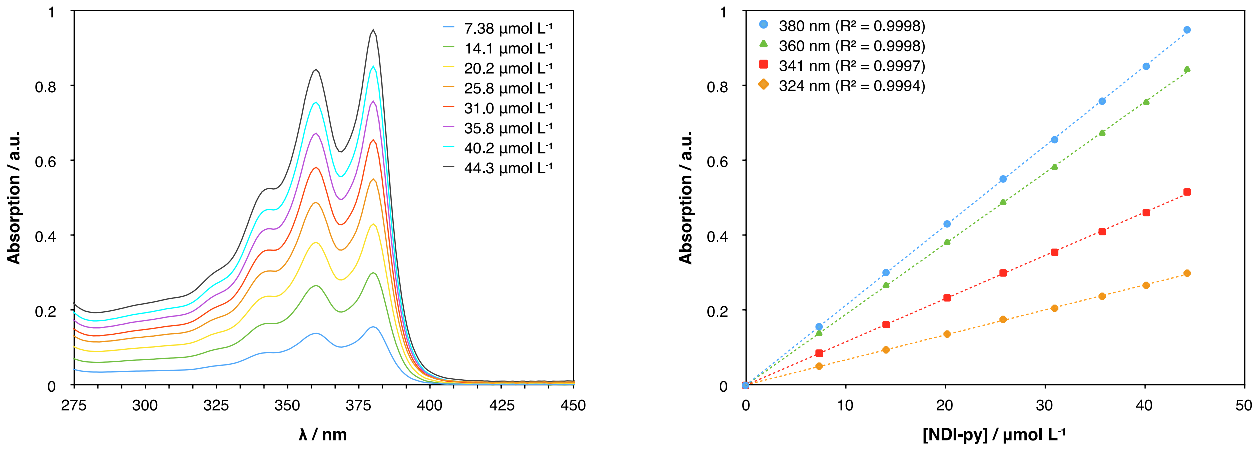

Supplementary Figure 15 | UV-Vis spectra and molar absortivities for NDI-py. Electronic spectra of NDI-py in DMF at several concentrations. Molar absorption coefficients (ε) were calculated at 380 nm (ε=2.1 x10^4 L mol^{-1} cm^{-1}), 360 nm (ε=1.9 x10^4 L mol^{-1} cm^{-1}), 341 nm (ε=1.1 x10^4 L mol^{-1} cm^{-1}) and 324 nm (ε=0.66 x10^4 L mol^{-1} cm^{-1}), in the 0.2 – 0.8 absorptivity window.

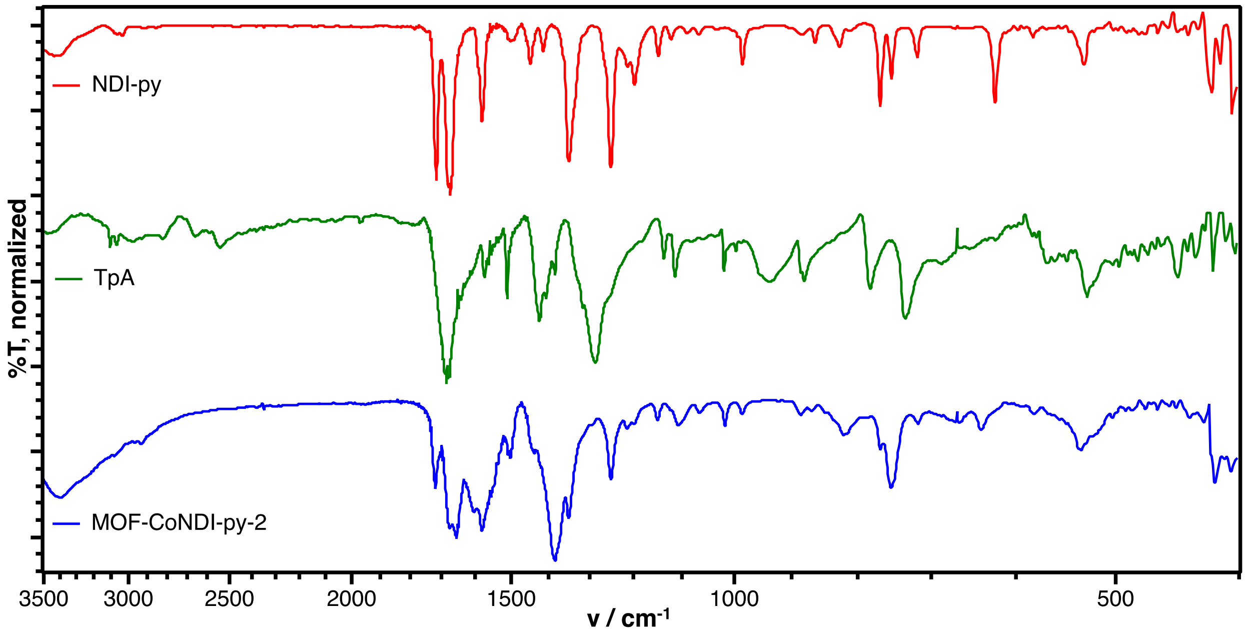

Supplementary Figure 16 | MOF FTIR spectra. Fourier-transform vibrational spectra of TpA, NDI-py and MOF-CoNDI-py-2 using KBr pellets. Samples were treated in an oven at 100 ºC for 3 h before analysis.

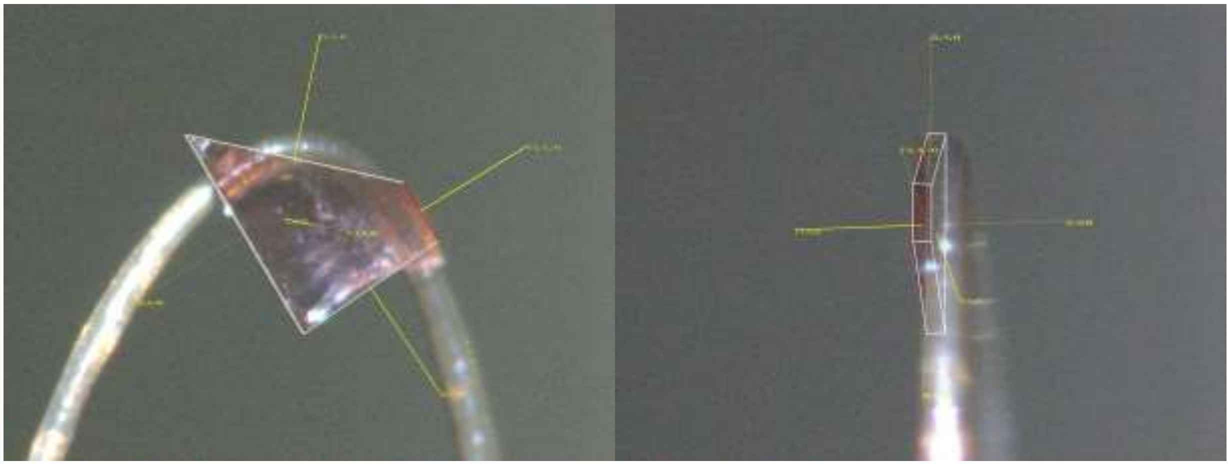

Supplementary Figure 17 | Crystal facet indexes. Crystal facet indexation images of MOF-CoNDI-py-2.

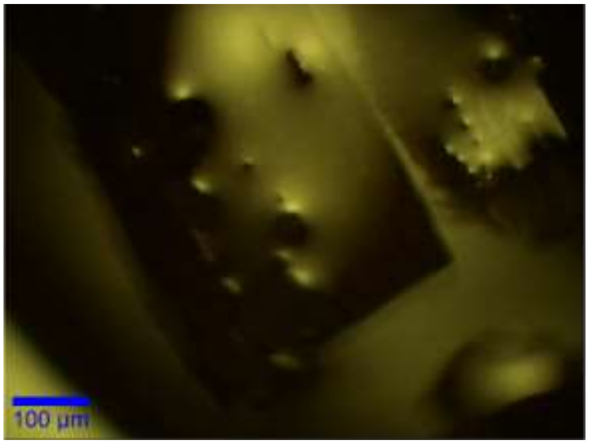

Supplementary Figure 18 | Crystal image. Image of the crystal analysed in the polarised Raman spectra.

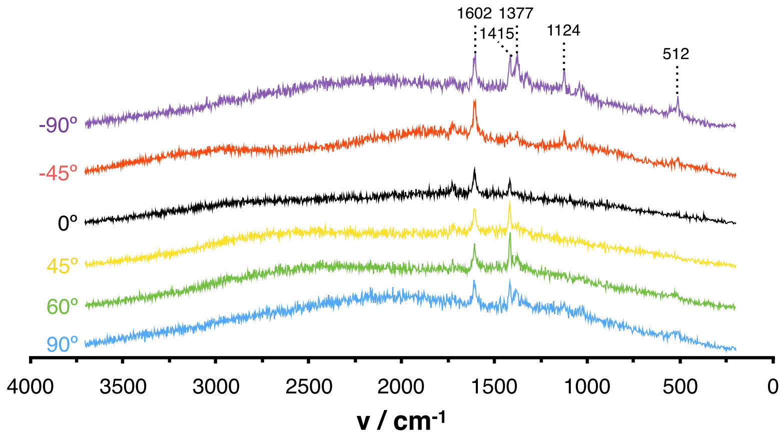

Supplementary Figure 19 | Polarized Raman spectra. Polarised confocal Raman spectra of MOF-CoNDI-py-2 at different incident and detector polariser angles.

Supplementary Table 1 Bond-Valence Sums parameters and calculations with the parameters from Supplementary Ref. 4. [a]

**Atom *i***	**Atom *j***	d_{ij} **(Å)**	R_{ij} [b]	v_{ij}	$\sum V_{ij}$ [c]
Co(1)	O	2.063	1.692	0.3668	
Co(1)	O	2.073	1.692	0.3573	
Co(1)	O	2.149	1.692	0.2909	**2.03**
Co(1)	O	2.063	1.692	0.3668	
Co(1)	O	2.073	1.692	0.3573	
Co(1)	O	2.149	1.692	0.2909	
Co(2)	O	2.026	1.692	0.4055	
Co(2)	O	2.029	1.692	0.4018	
Co(2)	N[b]	2.077	1.840	0.5273	
Co(2)	O	2.153	1.692	0.2875	**2.11**
Co(2)	N[b]	2.162	1.840	0.4191	
Co(2)	O	2.189	1.692	0.2800	
Co(2)	O	2.163	1.692	0.2612	

[a] Using the equation $\sum e_{ij} = V_{ij}$; $v_{ij} = e^{(R_{ij}-d_{ij})/b}$.
[b] Parameters from Supplementary Ref. 4.
[c] Applied a factor of 0.5 to take thermal movements into account.

Supplementary Table 2 | Reported responsivities. Responsivities in other materials, reported in the literature, to compare to MOF-CoNDI-py-2.

**Material**	**Responsivity (A W^{-1})**	**Reference**
Si	≈1	Chemical Society Reviews 44(11):3691-3718 (2015); Ref. 5
InGaAs	≈1	Chemical Society Reviews 44(11):3691-3718 (2015); Ref. 5
In_2Se_3	≈10^2	ACS Nano 8, 514 (2014); Ref. 6
MoS_2	≈10^3	Advanced Materials, 25, 3456 (2013); Ref. 7
TiS_3	≈10^3	Advanced Optical Materials 2, 641 (2014); 8
GaTe	≈10^4	ACS Nano 8, 752 (2014); Ref. 19
**MOF-CoNDI-py-2**	**≈10^5**	**This work**
MoS_2–PbS	≈10^6	Adv. Mater. 27, 176 (2014); Ref. 10
Graphene–MoS_2	≈10^7	Scientific Reports 4, Article number: 3826 (2014); Ref. 11
Graphene–PbS	≈10^8	Nature Nanotechnology 7, 363–368 (2012); Ref. 12

Supplementary Table 3 | Crystallographic data. Crystal data and refinement parameters for MOF-CoNDI-py-2 CCDC 1517923 contain the supplementary crystallographic data for this paper. These data can be obtained free of charge from The Cambridge Crystallographic Data Centre.

Parameter	MOF-CoNDI-py-2
Empirical formula	$C_{27}H_{19.5}Co_{1.5}N_3O_{9.25}$
Molecular weight	622.35 g mol^{-1}
Temperature	100(2) K
Crystal system	Monoclinic
Space group	C2/c
a	46.903(3) Å
b	9.5709(7) Å
c	17.9079(13) Å
α	90.00º
β	94.066(3)º
γ	90.00º
Cell volume	8018.7(10) Å^3
Z, Z'	8, 0
ρ (calc.)	1.031 g cm^{-3}
μ	0.668 mm^{-1}
F(000)	2536.0
Radiation	MoKα, 0.71075 nm
2Θ range	4.344º to 54.966º
Index ranges	$-60 \le h \le 47$, $-12 \le k \le 11$, $-23 \le l \le 23$
Reflections collected	23715
Independent reflections	8944 [R_{int} = 0.0696, R_σ = 0.0943
Data / restraints / parameters	8944 / 264 / 491
Goodness-of-fit on F^2	1.237
Final R indexes [I' ≥ 2σ(I)]	R_1 = 0.1195, wR_2 = 0.3223
Final R indexes [all data]	R_1 = 0.1862, wR_2 = 0.3662
Largest diff. peak/hole / e Å^{-3}	1.67 / –0.93

Supplementary Table 4 | Lamp output intensity. Lamp output intensities in the photoconductivity measurements.

**λ (nm)**	**Output (pW mm^{-2})**
350	31
380	40
460	54
510	49
550	47
630	41
700	9
800	7

Supplementary Note 1: Fourier-Transformed Infrared spectra (FTIR)

The infrared spectrum of MOF-CoNDI-py-2 was interpreted as a sum of its components' individual infrared spectra with a few notable changes. It was compared with NDI-py, and similar compounds[13], TpA anion[14] spectra, DMF[15] and nitrate anion (as a potassium salt, KNO_3) data[16] . Most peaks remain unchanged and the only noticeable variations are in frequencies of the coordinated groups, namely: i) the in-plane C–N–C scissors of NDI-py (from 623 cm^{-1} to 639 cm^{-1}); ii) asymmetrical O=C=O stretching of TpA (from 1617 cm^{-1} to 1654 cm^{-1}); iii) a second asymmetrical O=C=O stretching of TpA (from 1558 cm^{-1} to 1606 cm^{-1}); iv) the O=C=O rocking of TpA (from 508 cm^{-1} to 533 cm^{-1}); and, possibly, v) the symmetrical O=C=O stretching of TpA (from 1397 cm^{-1} to 1383 cm^{-1}), which is superimposed with a nitrate N–O stretching and it is hard to precisely determine its position.

FTIR data analysis showed that TpA suffers deprotonation – in agreement with crystallographic data – and also detected the presence of guest molecules such as water. DMF bands (2930 cm^{-1}, 1677 cm^{-1}, 1502 cm^{-1} and 1437 cm^{-1}) are superimposed with other bands, so it is not possible to confirm its presence.

Supplementary Note 2: Polarised Raman spectra

Polarised confocal Raman spectra of MOF-CoNDI-py-2 were acquired by moving the angle between incident and detector polarisers on a suitable MOF-CoNDI-py-2 crystal, shown in Figure 9. Sets of data were collected by fixing the incident polariser at 0º and varying the detector polariser at –90º, –45º, 0º, 45º, 60º and 90º relative to the detector. These spectra are shown in Supplementary Figure 19.

The spectra suffer from background sample fluorescence. A simple analysis indicates that by varying the angle between incident and detector polarisers in the Raman spectra for a MOF-CoNDI-py-2 crystal it can be seen the bands at 512 cm^{-1}, 1124 cm^{-1}, and 1377 cm^{-1}, are mostly influenced by the varying angle, while the bands at 1415 cm^{-1}, and 1602 cm^{-1}, suffer small influence. This observation is in agreement to the anisotropic nature of these crystals.

Supplementary References

1. Vinck, E., Carter, E., Murphy, D. M. & Doorslaer, S. V. Observation of an Organic Acid Mediated Spin State Transition in a Co(II)−Schiff Base Complex: An EPR, HYSCORE, and DFT Study. *Inorg. Chem.* **51**, 8014–8024 (2012).
2. Pietrzyk, P., Srebro, M., Radoń, M., Sojka, Z. & Michalak, A. Spin Ground State and Magnetic Properties of Cobalt(II): Relativistic DFT Calculations Guided by EPR Measurements of Bis(2,4-acetylacetonate)cobalt(II)-Based Complexes. *J. Phys. Chem. A* **115**, 2316–2324 (2011).
3. Katsuta, S. *et al.* Synthesis of pentacene-, tetracene- and anthracene bisimides using double-cyclization reaction mediated by bismuth(III) triflate. *Chem. Commun.* **47**, 10112–10114 (2011).
4. Brese, N. E. & O'Keeffe, M. Bond-Valence Parameters for Solids. *Acta Cryst.* **B47**, 192–197 (1991).
5. Buscema, M. *et al.* Photocurrent generation with two-dimensional van der Waals semiconductors. *Chem. Soc. Rev.* **44**, 3691–3718 (2015).
6. Jacobs-Gedrim, R. B. *et al.* Extraordinary Photoresponse in Two-Dimensional In_2Se_3 Nanosheets. *ACS Nano* **8**, 514–521 (2014).
7. Zhang, W. *et al.* High-Gain Phototransistors Based on a CVD MoS_2 Monolayer. *Adv. Mater.* **25**, 3456–3461 (2013).
8. Island, J. O. *et al.* Ultrahigh Photoresponse of Few-Layer TiS_3 Nanoribbon Transistors. *Adv. Opt. Mater.* **2**, 641–645 (2014).
9. Liu, F. *et al.* High-Sensitivity Photodetectors Based on Multilayer GaTe Flakes. *ACS Nano* **8**, 752–760 (2014).
10. Kufer, D. *et al.* Hybrid 2D–0D MoS_2–PbS Quantum Dot Photodetectors. *Adv. Mater.* **27**, 176–180 (2014).
11. Zhang, W. *et al.* Ultrahigh-Gain Photodetectors Based on Atomically Thin Graphene-MoS_2 Heterostructures. *Sci. Rep.* **4** (2014).
12. Konstantatos, G. *et al.* Hybrid graphene–quantum dot phototransistors with ultrahigh gain. *Nat. Nanotechnol.* **7**, 363–368 (2012).
13. Refat, M.S., Killa, H.M.A., Grabchev, I. & El-Sayed, M.Y. Synthesis and characterization of N,N'-bis[2-hydroxyethyl]-1,4,6,8-naphthalenediimide with para substituted of phenols based on charge-transfer complexes. *Spectrochim. Acta* **68**, 123–133 (2007).
14. Varghese, H. T., Panicker, C. Y., Philip, D., Sreevalsan, K. & Anithakumary, V. IR, Raman and SERS spectra of disodium terephthalate. *Spectrochim. Acta* **68**, 817–822 (2007).
15. Jones, R. L. The Infrared Spectra of Some N-Substituted Amides in the Vapor State. *J. Mol. Spec.* **11**, 411–421 (1963).
16. http://sdbs.riodb.aist.go.jp. Spectral Database for Organic Compounds of the National Institute of Advanced Industrial Science.
